# Biomarker potential of vanin-1-derived pantothenic acid in diabetes and its associated cardiovascular complications

**DOI:** 10.1038/s41598-025-19271-5

**Published:** 2025-09-15

**Authors:** Nada K. Shoura, Mahmoud H. Elbatreek, Mayada M. Mousa, Hany M. El-Bassossy, Omar Y. El-Azzazy

**Affiliations:** 1 Department of Pharmacy Practice, Faculty of Pharmacy, Zagazig University, Zagazig, Egypt; 2 Department of Pharmacology and Toxicology, Faculty of Pharmacy, Zagazig University, Zagazig, Egypt; 3 Cedars Sinai Medical Center, Los Angeles, CA 90048 USA; 4 Department of Internal Medicine, Faculty of Medicine, Zagazig University, Zagazig, Egypt; 5 Clinical Pharmacy Program, Zagazig National University, 10th of Ramadan City, Zagazig, Egypt

**Keywords:** Cardiovascular disease, Diabetes, Obesity, Pantothenic acid, Vanin-1, Cardiovascular diseases, Endocrine system and metabolic diseases

## Abstract

The enzyme vanin-1 produces pantothenic acid (PA) and cysteamine, but the role of the vanin-1 /PA axis in metabolic and cardiovascular diseases remains less explored than the vanin-1 /cysteamine pathway. This study investigated the relationship between vanin-1 and PA with obesity, type-2 diabetes (T2D), and related cardiovascular complications (CVD). We found that patients with T2D, with or without CVD, had significantly lower plasma PA levels, which negatively correlated with glycemic markers and positively with HDL cholesterol. Individuals in the lowest PA tertile exhibited a markedly higher prevalence of T2D (76.6%). While vanin-1 levels did not significantly differ between groups, a trend towards higher levels in T2D was observed, and higher vanin-1 correlated with higher HbA1c, with the highest vanin-1 tertile showing a greater proportion of T2D (62.5%). Importantly, low PA levels were significantly associated with increased odds of T2D in patients with obesity (OR = 7.61) and increased odds of diabetes with CVD in both patients with and without obesity (OR = 12.03, OR = 7.24, respectively). In contrast, high vanin-1 was not associated with increased odds. Our findings suggest that low PA levels may serve as a potential biomarker for T2D and T2D with CVD. Future research should investigate the impact of vanin-1 modulation on PA levels and the therapeutic potential of PA supplementation in managing these conditions.

**Supplementary Information:**

The online version contains supplementary material available at 10.1038/s41598-025-19271-5.

## Introduction

Diabetes mellitus (DM) is a chronic metabolic disorder characterized by persistent hyperglycemia, which arises from impaired insulin secretion, diminished insulin action, or a combination of both mechanisms^1^. The prevalence of DM is rapidly increasing worldwide, paralleling the alarming rise in obesity rates. Both conditions have reached epidemic proportions, significantly impacting public health and clinical outcomes^2,3^.Notably, at least half of individuals with diabetes and obesity face a heightened risk of developing cardiovascular diseases (CVD). This condition contributes to a significant portion of their mortality, estimated to be between one-third to one-half^4,5^. These concerning statistics underscore the urgent need for effective management strategies to prevent diabetes-related cardiovascular complications, beyond merely achieving glycemic control to delay the onset of these outcomes^6^.

**Fig. 1:**
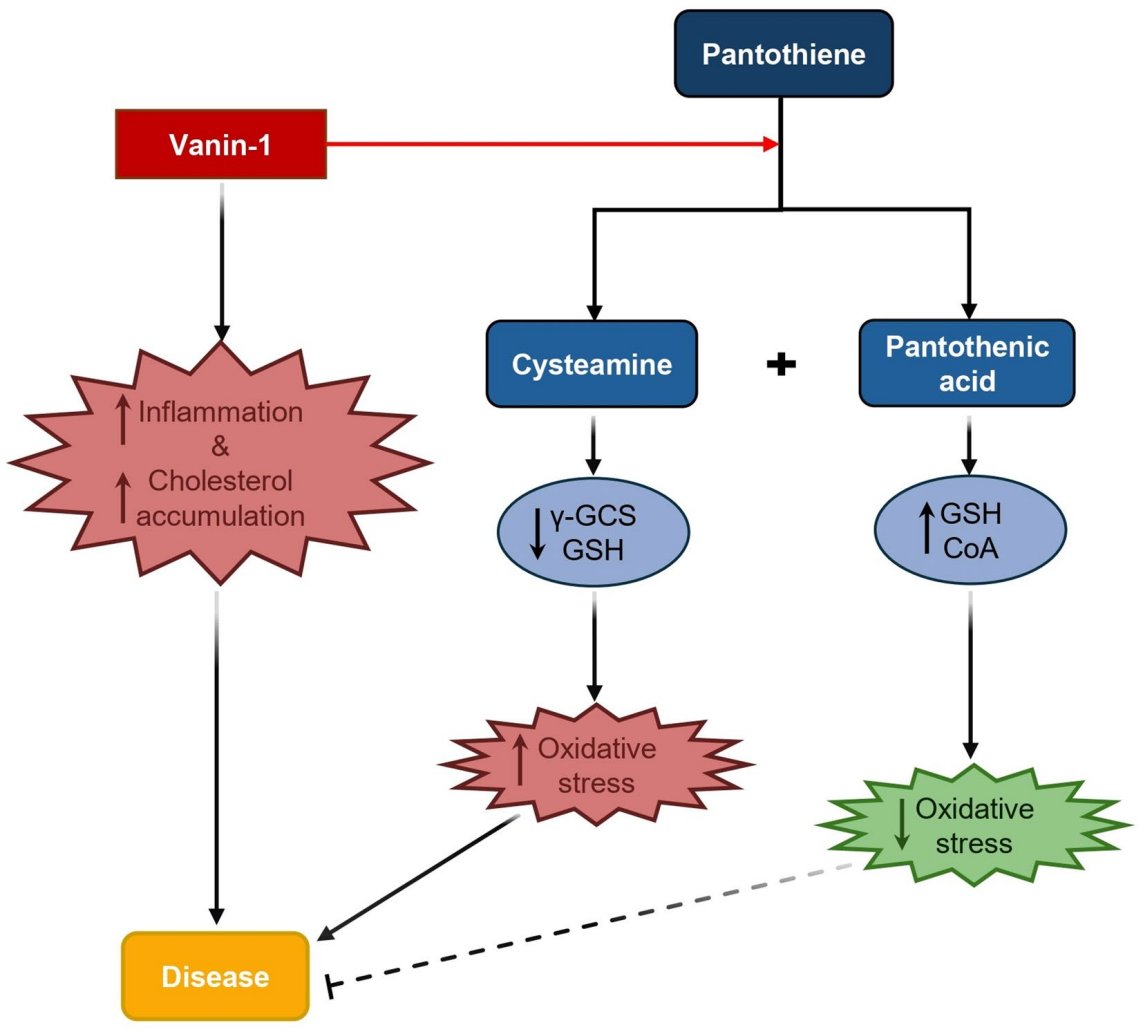
Schematic overview of vanin-1’s role in disease. CoA, coenzyme A; γ-GCS, γ- glutamylcysteine synthetase; GSH, glutathione.

Vascular non-inflammatory molecule-1 (vanin-1) is a membrane-bound enzyme with pantetheinase activity, catalyzing the hydrolysis of pantetheine to produce pantothenic acid (vitamin B5, PA) and cysteamine^7^. Elevated levels of vanin-1 have been associated with increased risks of diabetes, obesity, and coronary artery disease^8–10^. Recent investigations have illuminated vanin-1’s role in oxidative stress regulation, particularly its influence on glutathione (GSH) levels, positioning it as a critical player in redox homeostasis^11^. Notably, high concentrations of cysteamine can inhibit γ-glutamylcysteine synthetase (γ-GCS), leading to depleted intracellular GSH stores and exacerbating oxidative stress-related conditions^12,13^. Conversely, PA is believed to exert antioxidant effects by promoting GSH synthesis and enhancing coenzyme A (CoA) availability, thereby mitigating oxidative stress^14–16^. The differing effects of vanin-1’s downstream products have created ambiguity regarding its involvement in various diseases, particularly as research has predominantly focused on the vanin-1/cysteamine pathway while neglecting the vanin-1/PA axis (Fig. 1, Schematic overview of vanin-1’s role in disease).

Numerous studies have demonstrated diverse associations between PA levels and diabetes^17–20^. Experimental evidence supports that PA and its precursor, dexpanthenol, exhibit protective properties across various tissues due to their anti-inflammatory, antioxidant, and anti-apoptotic effects^21–23^. Crucially, these compounds have shown promise in safeguarding cardiac function against the adverse effects of diabetes and preserving pancreatic β-cell integrity in the face of oxidative stress^24,25^. While direct clinical data is scant, emerging data suggest a link between low PA levels and cardiovascular diseases; for instance, lower plasma PA levels have been reported in patients with coronary heart disease^26^. Furthermore, pantethine treatment (a PA derivative) showed efficacy in lowering cardiovascular disease risk markers such as total cholesterol and low-density lipoprotein cholesterol (LDL-C) in patients with low to moderate CVD risk^27^. The relationship between PA and obesity is complex; while some studies indicate that higher PA intake may reduce obesity risk, others suggest an association with increased obesity likelihood^28,29^.

In this study, we aim to evaluate the biomarker potential of vanin-1 and PA together in the context of obesity, type-2 diabetes, and diabetes-related cardiovascular complications. By investigating both markers simultaneously, we seek to elucidate their combined roles in metabolic health and to identify potential therapeutic targets for managing these interconnected conditions, addressing a significant gap in the literature where prior research has typically focused on one of these factors in isolation.

## Methods

### Study design and setting

This cross-sectional case-control study was performed between November 2022 and March 2023 at the Department of Internal Medicine of Zagazig University Hospitals. The study protocol was approved by the faculty of medicine of Zagazig University institutional review board (approval number 9350-23-2-2022) before subjects enrollment. The study was performed in accordance with the ethical standards laid down in the 1964 Declaration of Helsinki. Each participant gave informed consent before their inclusion in the study. Our study consists of four groups (35 subjects each): healthy controls, patients with obesity without diabetes, patients with diabetes, and patients with diabetes with cardiovascular disease (CVD).

Patients with diabetes, and patients with diabetes with CVD were recruited from diabetes and endocrine inpatient unit and the cardiology unit, respectively, while healthy controls and patients with obesity without diabetes were recruited voluntarily from the general population through local advertisements and word of mouth. Our study exclusion criteria were age less than 18 years old or more than 65 years old, pregnancy, type 1 diabetes, malignant tumors, severe renal dysfunction (defined as estimated glomerular filtration rate < 15 mL/min/1.73 m2), and history of chronic liver disease.

Diabetes was defined as a fasting plasma glucose level of ≥ 126 mg/dL or HbA1c of ≥ 48 mmol/mol according to American Diabetes Association criteria^30^ or on current treatment with insulin and/or oral hypoglycemic agents. Myocardial ischemia was defined as presenting with acute coronary syndrome (ACS) or a history of stable or unstable angina, which was confirmed by a cardiac enzyme test, ECG, and echocardiography. Heart failure was defined as a history of chronic heart failure confirmed by Natriuretic Peptide Tests (BNP), ECG, and Echocardiography. Cardiomyopathy was defined as a history of early stage left ventricular remodeling and diastolic dysfunction preceding overt heart failure, confirmed by Natriuretic Peptide Tests (BNP), ECG, and Echocardiography. Obesity was defined as BMI > 30 kg/m2.

### Data collection

We used case record forms completed by trained medical professionals to collect detailed information on each subject’s medical history, smoking habits, and medications intake. Medical histories were documented based on thorough clinical evaluations, diagnostic findings from patient records, and structured interviews. Weight and height were recorded, and body mass index (BMI, kg/m²) was calculated. Demographic data and laboratory values were retrieved from the hospital medical records; We used the data within 1 week of the sample collection. Fasting blood glucose was measured after an overnight fast of at least 8 h, and postprandial blood glucose was measured 2 h after a standardized meal using venous blood samples analyzed in the hospital’s central laboratory.

### Blood sample collection and biomarkers analysis

Blood samples of all participants were collected in a heparin-coated tube after fasting for 8 h. Samples were centrifuged at 6000 rpm for 15 min at room temperature, plasma was divided into four Eppendorf tubes and kept at -80 until analysis. Vanin-1 plasma levels were measured using commercially available enzyme-linked immunosorbent assay Kits (ELISA) ((FineTest®, China) which has a reported sensitivity of 0.094 ng/mL and demonstrate high specificity, with no significant cross-reactivity or interference with related analogues. The intra- and inter-assay coefficients of variation (CVs) were < 8% and < 10%, respectively. PA level was measured using commercially available ELISA kits ((BT Lab bioassay, China) with a sensitivity of 10.96 ng/mL, with intra- and inter-assay CVs also reported to be < 8% and < 10%, respectively. Reduced Glutathione (GSH) was measured using a commercial colorimetric kit (Elabscience, USA). Malondialdehyde (MDA) level was measured using the thiobarbituric acid test method with a commercial colorimetric kit (Biodiagnostic, Egypt). All assays were performed following the manufacturers’ instructions.

### Statistical analysis

All statistical analyses were performed using SPSS for Windows software (ver. 25). Continuous variables were expressed as a mean $\:\pm\:$ standard deviation or median (Interquartile range), while categorical variables were expressed as the number and percentage (%). The normal distribution of the continuous variables was tested using the Shapiro–Wilk test before deciding the appropriate statistical test. For parametric variables, one-way ANOVA with the Fisher’s Least Significant Difference (LSD) post hoc test was used to evaluate the difference between the groups. For nonparametric variables (vanin-1, PA, GSH, MDA, and Triglycerides levels), the Kruskal Wallis test followed by pairwise comparison was used to evaluate the difference between the groups. The chi-square test (X^2^) and Montecarlo exact test were used for categorical variables. The correlations between variables were examined using Spearman’s correlation test. We performed multinomial logistic regression analysis adjusted for age, sex, and BMI to calculate Odds ratios (ORs) and their corresponding 95% CIs to investigate the association of plasma vanin-1 and PA levels with the development of diabetes, diabetes cardiovascular complications, and obesity. The tertiles of vanin-1 levels and PA of the subjects included in the study were used to classify these subjects into three groups. We considered subjects in the vanin-1 third tertile (vanin-1 level > 1.574 ng/mL) to have a high vanin-1 level, and subjects in the PA first tertile (PA level < 850.27 ng/ml) to have a low PA level. Differences with *p* < 0.05 were considered statistically significant.

## Results

### Patient characteristics and anthropometric data

The clinical and anthropometric characteristics of the study are summarized in Table 1. No significant differences were observed among the groups with respect to demographic data. However, a statistically significant difference in BMI was noted, with the control group exhibiting the lowest BMI. The diabetes groups demonstrated significantly elevated levels of HbA1c, fasting blood glucose, postprandial blood glucose, and triglycerides, alongside notably lower levels of HDL cholesterol. No significant differences were found between the groups regarding total cholesterol, LDL cholesterol, or MDA levels. Among the patients with cardiovascular diseases, 62.5% had ischemic heart disease, including acute coronary syndrome and stable angina (*n* = 20), 28.2% had chronic heart failure (*n* = 9), and 9.3% had cardiomyopathy (*n* = 3).

**Table 1:** Clinical characteristics and demographic information.

	Control	Obesity	Diabetes	Diabetes with CVD	*P* value
	Mean ± SD	Mean ± SD	Mean ± SD	Mean ± SD	
Demographic data					
Age (year)	51.84 ± 7.805	52.97 ± 7.698	49.78 ± 8.798	51.66 ± 7.23	0.654
BMI (kg/m^2^)	25.87 ± 2.6	38.09 ± 5.98 †	30.97 ± 4.25 †‡	34.02 ± 7.82 †‡	< 0.001
Female (%)	19 (59.4%)	16 (50%)	19 (59.4%)	16 (50%)	0.769
Smoker (%)	6 (18.7%)	5 (15.6%)	4 (12.5%)	7 (21.9%)	0.778
Laboratory data					
HbA1c (%) (mmol/mol)	5.15 ± 0.4 31.03 ± 2.48	5.51 ± 0.46 34.16 ± 2.85	8.84 ± 2.2 †‡ 54.8 ± 13 †‡	8.91 ± 2.18 †‡ 55.24 ± 13.51 †‡	< 0.001
FBG (mg/dl)	91.73 ± 12.94	92.25 ± 16.64	196.34 ± 86.53 †‡	186.31 ± 47.22 †‡	< 0.001
PPBG (mg/dl)	116.78 ± 9.96	116.81 ± 12.34	265.09 ± 110.01 †‡	267.63 ± 71.46 †‡	< 0.001
TC (mg/dl)	153.53 ± 37.2	165.66 ± 29.15	169.41 ± 50.75	166.43 ± 60.84	0.531
HDL (mg/dl)	48.78 ± 13.17	42.38 ± 11.99	33.11 ± 13.4 3 †‡	35.38 ± 11.54 ‡	< 0.001
LDL (mg/dl)	81.08 ± 38.98	96.71 ± 33.56	87.31 ± 38.32	98.5 ± 48.32	0.263

	Median (IQR)	Median (IQR)	Median (IQR)	Median (IQR)	*P* value
TG (mg/dl)	90(76 − 119)	101.5(83.5–172)	146(111.88–214.4) †‡	152.5(96 − 246) †	< 0.001
MDA (nmol/ml)	10.76(8.87–12.69)	11.08(8.15–12.86)	12.38(9.53–16.47)	12.43(9.66–15.98)	0.083
GSH (mg/L)	3.78(2.86–9.81)	3.62(2.76–4.97)	4.22(2.76–5.232)	4.56(2.54–5.64)	0.8963
Medications, n (%)					
Insulin	-	-	15 (48.4%)	16 (50%)	0.802
Metformin	-	-	13(40.6%)	16(50%)	0.451
Sulfonylureas	-	-	12(37.5%)	15(46.9%)	0.448
DPP-4 inhibitors	-	-	9(28.1%)	10(31.3%)	0.784
SGLT2 inhibitors	-	-	2(6.3%)	6(19.4%)	0.148
Thiazolidinediones	-	-	1(3.1%)	2(6.3%)	> 0.999

† *p* < 0.05 vs. control, ‡ *p* < 0.05 vs. obesity. BMI, body mass index; GSH, glutathione; HbA1c, glycated hemoglobin; FBG, fasting blood glucose; PPBG, Postprandial Blood Glucose; HDL, high-density lipoprotein; IQR, interquartile range, LDL, low-density lipoprotein; MDA, malondialdehyde; TC, total cholesterol; TG, triglycerides.

### Low pantothenic acid levels represent a biomarker in diabetes

**Fig. 2:**
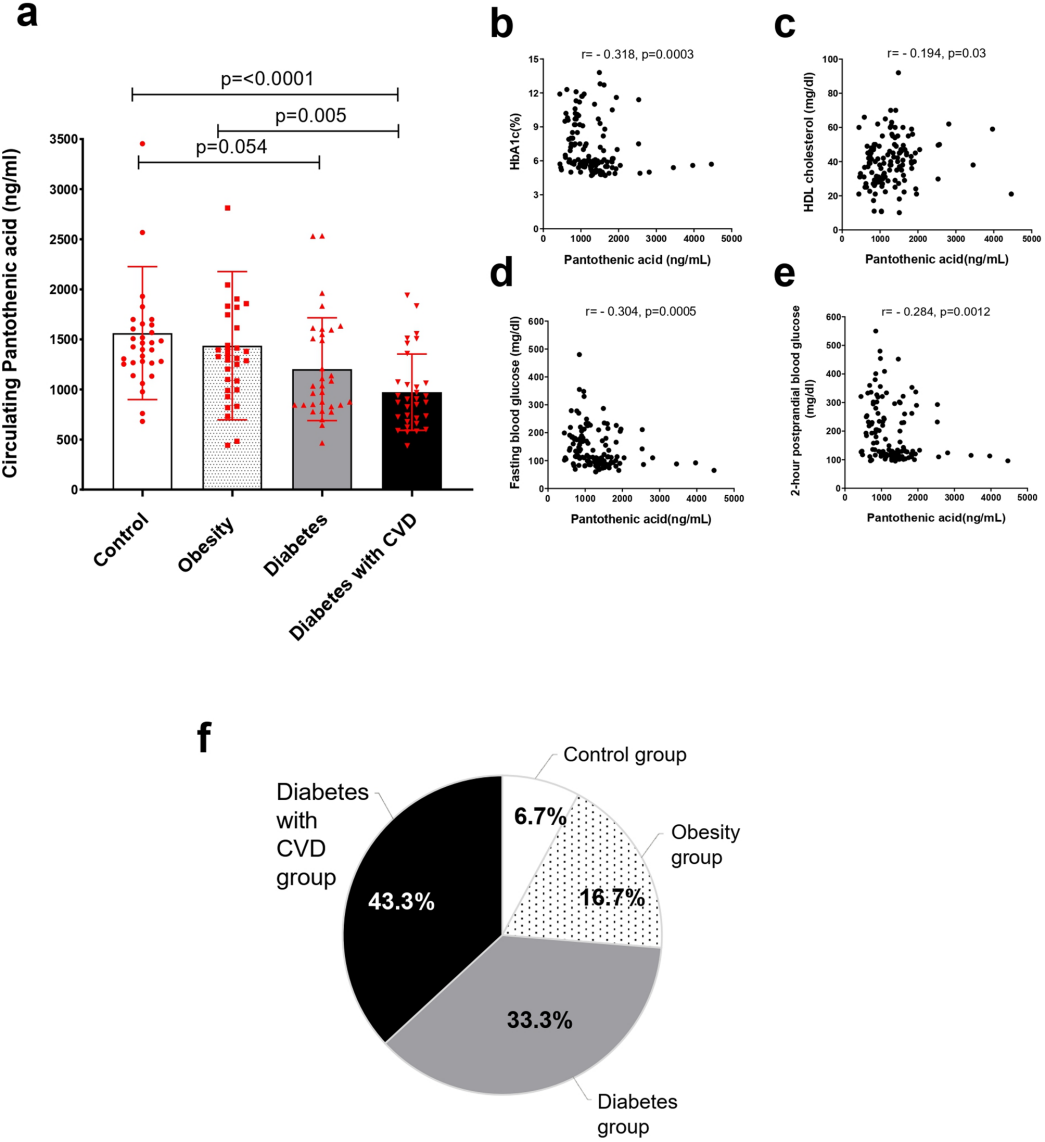
Pantothenic acid levels are reduced in diabetes. **a**, Comparison between the studied groups regarding serum pantothenic acid, data are expressed as the mean and standard deviation. **b**, Correlation between plasma pantothenic acid and glycated hemoglobin. **c**, Correlation between plasma pantothenic acid and fasting blood glucose. **d**, Correlation between plasma pantothenic acid and postprandial blood glucose. **e**, Correlation between plasma pantothenic acid and HDL-cholesterol, r Spearman rank correlation coefficient. **f**, Study participants distribution within pantothenic acid Q1 tertile (< 850.27 ng/ml).

Plasma PA levels were significantly lower in patients with diabetes with CVD compared to both healthy controls and individuals with obesity. Additionally, PA levels in the diabetes group were lower than those in the control group (*p* = 0.05), as illustrated in Fig. 2a. To further elucidate the relationship between PA and other clinical parameters, we conducted a correlation analysis (Supplementary Table S1). A statistically significant negative correlation was observed between PA and HbA1c (*r* = -0.318, *p* = 0.0003) (Fig. 2b), fasting blood glucose (*r* = -0.304, *p* = 0.0005) (Fig. 2d), and postprandial blood glucose (*r* = -0.284, *p* = 0.0012) (Fig. 2e). Conversely, a significant positive correlation was found between PA and HDL cholesterol (*r* = 0.194, *p* = 0.03) (Fig. 2c). Notably, 76.6% (*n* = 23) of the subjects in the lowest tertile of pantothenic acid (*n* = 30) were diagnosed with diabetes (Fig. 2f). These findings suggest that higher PA levels are associated with favorable metabolic markers, while lower PA levels correlate with adverse diabetes-related indicators.

### Vanin-1 levels show a trend toward elevation in diabetes

**Fig. 3:**
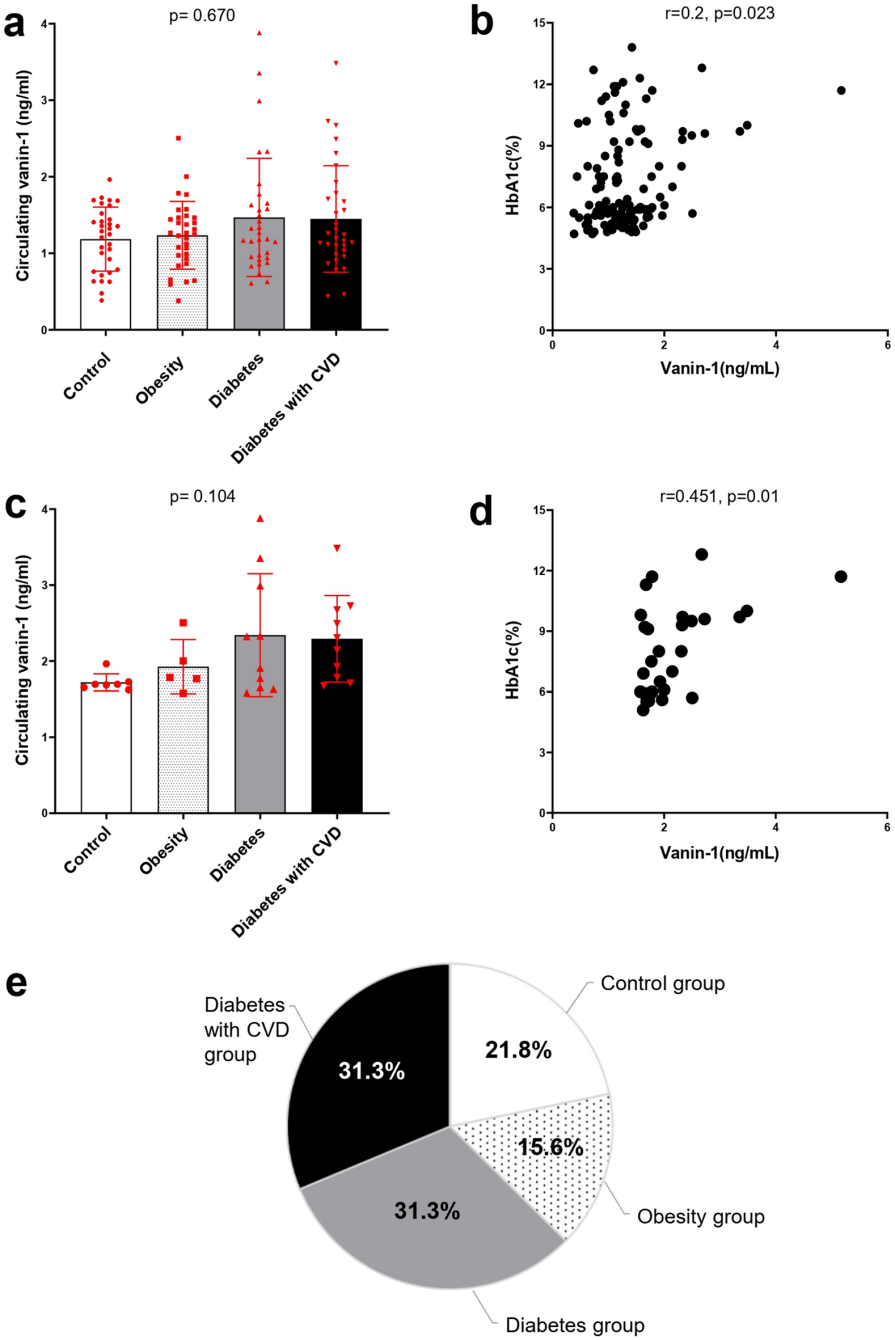
High Vanin-1 levels are dominant in diabetes. **a**, Comparison between the studied groups regarding plasma vanin-1, data are expressed as the mean and standard deviation. **b**, Correlation between plasma vanin-1 and glycated hemoglobin, r Spearman rank correlation coefficient. **c**, Comparison between the studied groups regarding serum Vanin-1 in vanin-1 Q3 tertile, data are expressed as the mean and standard deviation. **d**, Correlation between plasma vanin-1 and glycated hemoglobin in vanin-1 Q3 tertile, r Spearman rank correlation coefficient. **e**, study participants distribution within vanin-1 Q3 tertile (> 1.574 ng/ml).

While vanin-1 levels did not vary significantly across the groups, there was a noticeable trend toward higher levels in the diabetes cohorts (Fig. 3a). To further investigate the role of vanin-1 in diabetes, we conducted a correlation analysis with the studied parameters (Supplementary Table S2). This analysis revealed a significant positive correlation between plasma vanin-1 and HbA1c (*r* = 0.2, *p* = 0.023) (Fig. 3b). Notably, the prevalence of diabetes in the highest tertile of vanin-1 (*n* = 32) was 62.5% (*n* = 20) (Fig. 3e).

When comparing plasma vanin-1 levels among different study groups within the third tertile (> 1.574 ng/mL), patients with diabetes exhibited a trend toward elevated vanin-1 levels (Fig. 3c). In this tertile, vanin-1 demonstrated a stronger positive correlation with HbA1c (*r* = 0.451, *p* = 0.01) (Fig. 3d).

### Oxidative stress markers show no significant differences across groups

Plasma levels of glutathione (GSH) and malondialdehyde (MDA) showed no statistically significant differences across the study groups (Table 1). Additionally, GSH showed no significant correlations with PA (Supplementary Table S1) or vanin-1 (Supplementary Table S2), except for a positive correlation with MDA (*r* = 0.223, *p* = 0.011) (Supplementary Table S3). Similarly, MDA showed no significant correlations with either PA (Supplementary Table S1) or vanin-1 (Supplementary Table S2).

### Low PA levels are associated with increased odds of obesity and diabetes

To assess the relationship between vanin-1, PA, and the odds of diabetes and related cardiovascular diseases, we conducted a multinomial logistic regression analysis (Table 2). Our findings revealed that low PA levels (< 850.27 ng/mL) were significantly associated with an increased odds of diabetes in patients with obesity (OR = 7.610, 95% CI = 1.37–42.05, *p* = 0.02). Additionally, low PA levels correlated with a higher odds of diabetes with cardiovascular disease (CVD) in both patients with/without obesity (OR = 7.237, 95% CI = 1.11–47.19, *p* = 0.038 for non-obese; OR = 12.028, 95% CI = 2.23–64.76, *p* = 0.004 for obese). In contrast, high vanin-1 levels did not demonstrate an association with increased odds for any of the conditions studied.

**Table 2:** Biomarkers levels and risk of disease status.

Disease status	Biomarkers level	OR (95% confidence interval)	*P*-value
Obesity	Vanin-1 > 1.574 ng/ml	0.637 (0.17–2.28)	0.489
	Pantothenic acid < 850.27 ng/ml	2.853 (0.51–15.99)	0.233
Diabetes in patients without obesity	Vanin-1 > 1.574 ng/ml	0.664 (0.11–3.81)	0.642
	Pantothenic acid < 850.27 ng/ml	5.124 (0.73–35.73)	0.099
Diabetes in patients with obesity	Vanin-1 > 1.574 ng/ml	2.146 (0.59–7.57)	0.235
	Pantothenic acid < 850.27 ng/ml	**7.610** ** (1.37–42.05)**	**0.02**
Diabetes with CVD in patients without obesity	Vanin-1 > 1.574 ng/ml	1.616 (0.363–7.19)	0.529
	Pantothenic acid < 850.27 ng/ml	**7.237** ** (1.11–47.10)**	**0.038**
Diabetes with CVD in patients with obesity	Vanin-1 > 1.574 ng/ml	1.326 (0.349–5.03)	0.679
	Pantothenic acid < 850.27 ng/ml	**12.028** ** (2.23–64.76)**	**0.004**

Significant values are in [bold].

CVD, cardiovascular disease; OR, odds ratio.

### Diabetes medications do not impact plasma vanin-1/PA levels

To evaluate the impact of various diabetes medications on vanin-1 and PA levels, we performed a binary logistic regression analysis (Supplementary Table S4). The results indicated that neither insulin therapy nor oral hypoglycemic agents were associated with decreased vanin-1 levels or increased PA levels. However, these findings should be interpreted with caution due to the small sample size and the possibility of polypharmacotherapy for most of the subjects; further large-scale studies are needed to draw more definitive conclusions.

## Discussion

To the best of our knowledge, there are few clinical studies that have investigated the association between vanin-1, PA, and diabetes along with its cardiovascular complications. Our findings indicate that patients with diabetes with cardiovascular comorbidities exhibit the lowest plasma levels of PA, and similarly low levels are observed in patients with diabetes overall. Furthermore, we identified a significant negative correlation between PA levels and various glycemic markers, with glycated hemoglobin demonstrating the strongest association. This suggests a potential link between reduced PA levels and glucose intolerance.

Notably, low PA levels were associated with a seven-fold increased likelihood of diabetes in patients with obesity, and an increased probability of diabetes with cardiovascular disease in both patients with (12-fold) and without obesity (7-fold). The striking prevalence of diabetes (76.6%) among subjects with low plasma PA levels further reinforces the association between reduced PA and diabetes, regardless of cardiovascular complications. It is worth noting that we further stratified diabetes based on obesity status since obesity is a common and metabolically significant comorbidity in diabetes, and its presence may influence both PA levels and their associations with different disease status. By analyzing patients with diabetes with and without obesity as separate subgroups, we aimed to explore potential differences in PA-related metabolic patterns, which may reflect distinct pathophysiological mechanisms. This approach provides a more refined understanding of PA’s relevance across varying metabolic profiles and may support future stratified therapeutic strategies.

The oxidative stress markers, GSH and MDA did not show significant differences across study groups or consistent correlations with vanin-1 or PA. These findings suggest that, within the context of our study population and measured parameters, systemic levels of GSH and MDA may not be the primary mediators of the observed associations between PA and diabetes with/ without CVD.

Our findings show lower PA levels in patients with diabetes with CVD compared to individuals with obesity, however, we did not observe a statistically significant difference in PA levels between the patients with obesity and patients with diabetes without CVD. The common metabolic and inflammatory state in obesity and diabetes might similarly suppress PA levels, obscuring group-level differences. Notably, the lower PA levels observed in diabetes with CVD group suggest a potential independent effect of CVD because of the additive influence of systemic chronic inflammation, endothelial dysfunction, or pharmacological treatments of CVD that alter PA levels.

In line with our findings, Gulle et al.^25^ demonstrated that dexpanthenol, a precursor to PA, alleviated diabetes-induced pancreatic damage and inflammation in rats, highlighting its antioxidant and potential antihyperglycemic properties. Also, a metabolomics study in China subsequently identified significantly lower levels of PA in patients with diabetes with nephropathy compared to patients with diabetes without complications^18^. Additionally, vanin-1-deficient animals displayed increased susceptibility to diabetes, suggesting impaired vanin-1 function may compromise tissue stress tolerance through reduced PA regeneration^31–33^. However, contrasting results emerged from Gogna et al., who found higher PA levels in patients with obesity with type 2 diabetes^17^. This discrepancy may be attributed to ethnic variations, as their study focused solely on South Indian Asian patients with diabetes.

In addition to its potential link to diabetes, PA may also play a protective role against cardiovascular complications associated with the condition. Supporting this hypothesis, Gulle et al. demonstrated that dexpanthenol could help prevent cardiovascular issues in diabetes by protecting endothelial function and reducing oxidative stress in a streptozotocin model of diabetes^25^. Similarly, Kalkan et al. showed that dexpanthenol administration significantly mitigated cardiac damage, improved ECG abnormalities, and lowered oxidative stress in a myocardial infarction model^34^. Recent studies have further confirmed the cardioprotective properties of vitamin B5 across various cardiac injury models by reducing oxidative stress and inflammation^35,36^. Consistent with our findings, a study from China reported that higher plasma levels of PA were linked to a lower incidence of coronary heart disease^10^. However, a conflicting study found a correlation between higher PA intake and increased coronary calcification scores in diabetes patients with chronic kidney disease^37^. However, they did not measure serum PA levels.

Regarding vanin-1, our findings indicated no significant differences in plasma levels among the study groups, though a trend toward higher levels was observed in patients with diabetes. Notably, vanin-1 levels demonstrated a stronger positive correlation with HbA1c in the highest tertile compared to the correlation in the whole dataset, as indicated by a higher correlation coefficient, suggesting a potential link between elevated vanin-1 and impaired glucose tolerance. However, we did not find an association between high vanin-1 levels and increased odds of obesity, diabetes, or diabetes with CVD. Supporting our findings, Küçük et al. reported higher serum vanin-1 levels in diabetes patients compared to those without diabetes, with a positive correlation to the insulin resistance index (HOMA-IR)^8^. Similarly, Hosohata et al. observed that the individuals with higher urinary vanin-1 had significantly higher HbA1c and plasma glucose levels^38^. Mosaad et al. reported a significant correlation between vanin-1 gene expression and HbA1c level^9^; however, our study assessed vanin-1 protein levels. Other studies did not find a significant correlation between vanin-1 and fasting blood glucose^10^ or HbA1c levels^8,10^.

These observations align with preclinical data indicating that vanin-1 serum levels are elevated in diabetes animal models. Specifically, knocking down vanin-1 in the liver alleviated insulin resistance and impaired glucose tolerance^39^. Mechanistically, vanin-1 may contribute to these issues by activating gluconeogenesis through the Akt signaling pathway^39,40^. However, while some studies noted increased vanin-1 activity in diabetic models, the absence of vanin-1 resulted in only mild improvements in glucose tolerance and insulin sensitivity^41^. Pharmacological inhibition of vanin-1 using RR6 also did not significantly affect insulin sensitivity or fasting blood glucose levels^42,43^. Interestingly, in models of vanin-1 deficiency, mice exhibited worsened insulin resistance and glucose tolerance. Yet, restoring vanin-1 activity in white adipose tissue significantly mitigated these effects, and enhancing vanin-1 expression in brown adipose tissue produced similar benefits^40,44^. The variability in findings across studies may stem from differences in assessing vanin-1 activity, suggesting that its effects on diabetes may be tissue-specific.

We did not find any previous studies examining the role of vanin-1 in cardiovascular complications of diabetes. However, there are some reports on the role of vanin-1 in cardiovascular diseases and cardiovascular disease risk factors. A recent study reported that patients with coronary artery disease had elevated plasma vanin-1 concentrations, which were linked to both the presence and severity of coronary artery disease^10^. Furthermore, vanin-1 deficiency leads to substantially reduced neointima formation following carotid artery ligation in animals^45^. Wedel et al.^46^ further confirmed these findings, they found that vanin-1 inhibition diminished the formation of neointima after aortic allografts, primarily by decreasing oxidative stress. Altogether, vanin-1 has an atherosclerotic effect, increasing the risk of cardiovascular diseases. Also, vanin-1 showed a positive correlation with several cardiovascular risk factors including waist circumference, BMI, and carotid intima-media thickness^8^.

Our present understanding of the vanin-1/PA role in metabolic diseases is ambiguous. As evident from our present study, this unclear understanding may arise from the differential role of vanin-1 and PA in diabetes and its associated CVD. While PA seems to be protective against these conditions, vanin-1 might have an unfavorable effect in these conditions. Moreover, previous studies have focused on the vanin-1/cysteamine arm of the pathway, which is thought to create a state of disease induction through participating in oxidative stress^7^, this perspective overlooked the vanin-1/ PA arm of the pathway which might hold promise in optimizing diabetes and its associated CVD management.

An intriguing finding of our study is the tendency for increased vanin-1 concentration alongside lower PA levels in diabetes. Given that vanin-1 catabolizes pantetheine to both PA and cysteamine, this apparent discrepancy suggests a more complex regulation of the pathway in diabetes. One possible explanation is a shift in the relative production of vanin-1 downstream products, potentially favoring cysteamine over PA. Preclinical studies have shown that cysteamine levels and vanin-1 activity can be elevated in certain disease states associated with oxidative stress, which is often increased in diabetes^9,12,13^. Additionally, altered Pantetheine availability due to alteration in Coenzyme A (CoA) metabolism in diabetes^47^, of which pantetheine is an intermediate, might contribute to low PA levels, since even with slightly increased vanin-1 activity, the net production of PA might still be reduced. Although current clinical studies directly addressing these mechanisms are limited, our findings underscore the need for future studies to simultaneously measure PA, cysteamine, pantetheine, and vanin-1 to provide a more comprehensive understanding of this complex pathway in the context of diabetes and its cardiovascular complications.

The primary contribution of this study is the investigation of associations between marker levels and various disease states as allowed by the cross-sectional case-control design of the study, thereby generating hypotheses for future research. However, this study has several limitations. First, the sample size in each group is relatively small. Second, the cross-sectional nature of the study limits our ability to establish causality and exclude the potential reverse causation between biomarkers and diseases, highlighting the need for larger interventional studies. Third, our study lacks a comparison group of patients with CVD without diabetes; the inclusion of such a group wasn’t feasible within the scope of our study due to ethical approval considerations. There is a crucial direction for future research to include such a group, with the necessary ethical approvals, to further elucidate the interplay between diabetes, CVD, and the investigated biomarkers. Additionally, we recognize that the lack of cysteamine measurements limits our ability to definitively determine the relative contributions of PA and cysteamine to the observed associations and to fully support the hypothesis of a dichotomic role of vanin-1. However, our decision to focus solely on PA levels was primarily was influenced by several factors (i) existing literature has indeed focused on the vanin-1/cysteamine axis, particularly its role in oxidative stress., while the vanin-1/PA pathway is often-overlooked ; (ii) we measured glutathione (GSH) levels, a key marker influenced by cysteamine, and found no significant differences in its level across our study groups or significant correlations with vanin-1, suggesting that the vanin-1/cysteamine axis might not be the primary driver of the associations observed; and (ii) our primary research question centered on the biomarker potential of PA in these conditions.

Future studies should prioritize the simultaneous measurement of both PA and cysteamine, alongside relevant markers of oxidative stress like GSH, to gain a more comprehensive understanding of the vanin-1 pathway’s role in metabolic and cardiovascular diseases and to fully explore the potential for a dichotomic mechanism. Lastly, as the study exclusively included Egyptian patients, further investigation is required to determine whether these findings are generalizable to other populations.

## Conclusions

In conclusion, this study highlights the significant association between low PA levels and the presence of diabetes, particularly in patients with cardiovascular complications. Our findings suggest that PA may serve as a potential biomarker for diabetes and its related conditions, with lower levels correlating with higher glycemic markers and a greater odds of diabetes in both individuals with and without obesity. While vanin-1 levels did not show significant differences between groups, a trend towards higher levels in patients with diabetes was observed, indicating a possible link to impaired glucose tolerance. These results underscore the need for further research to explore the mechanisms underlying the roles of PA and vanin-1 in diabetes and cardiovascular health, as well as to assess the generalizability of our findings across diverse populations. Future studies should aim to elucidate the clinical implications of PA supplementation and vanin-1 inhibition as potential therapeutic strategies in managing diabetes and its complications.

## Supplementary Information

Below is the link to the electronic supplementary material.

Supplementary Material 1

## Data Availability

All data generated or analyzed during this study are included in this published article and the supplementary material.

## Abbreviations

DM: Diabetes mellitus
CVD: Cardiovascular diseases
vanin-1: Vascular non-inflammatory molecule-1
GSH: Reduced glutathione
γ-GCS: γ-glutamylcysteine synthetase
CoA: Coenzyme A
BNP: Natriuretic Peptide Tests
BMI: Body mass index
ELISA: Enzyme-linked immunosorbent assay Kits
MDA: Malondialdehyde
PA: Pantothenic acid
ORs: Odds ratios
HDL: High-density lipoprotein cholesterol
LDL: Low-density lipoprotein cholesterol
HbA1c: Glycated hemoglobin
ECG: Electrocardiogram
HOMA-IR: Insulin resistance index
