# Supplementary material for: Biomarker potential of vanin-1-derived pantothenic acid in diabetes and its associated cardiovascular complications
Source: Sci Rep. 2025 Sep 15;15:32549. doi: 10.1038/s41598-025-19271-5 (PMC12436645; doi:10.1038/s41598-025-19271-5)
Supplement: Supplementary file 1 — Supplementary Material 1 [file 41598_2025_19271_MOESM1_ESM.docx]

***Supplementary Material***

**Biomarker Potential of Vanin-1-derived Pantothenic Acid in Diabetes and its Associated Cardiovascular Complications**

Nada K. Shoura^1^*, Mahmoud H. Elbatreek^23^, Mayada M. Mousa^4^, Hany M. El-Bassossy^25^, Omar Y. El-Azzazy^1^

^1^Department of Pharmacy Practice, Faculty of Pharmacy, Zagazig University, Zagazig, Egypt.

^2^Department of Pharmacology and Toxicology, Faculty of Pharmacy, Zagazig University, Zagazig, Egypt.

^3^Cedars Sinai Medical Center, Los Angeles, CA 90048

^4^Department of Internal Medicine, Faculty of Medicine, Zagazig University, Zagazig, Egypt.

^5^Clinical Pharmacy Program, Zagazig National University, 10^th^ of Ramadan City, Egypt.

***Correspondence to** Nada K. Shoura**; Email:** [nkshori@pharmacy.zu.edu.eg](mailto:nkshori@pharmacy.zu.edu.eg)**; Phone:** +201094909759.

**Supplementary Table S1. Correlation between** **plasma pantothenic acid and the studied parameters.**

|  | r | p |
| --- | --- | --- |
| Age (year) | .086 | .502 |
| BMI (kg/m^2^) | 0.024 | 0.852 |
| HbA1c (%) | **-0.318** | **0.0003** |
| Fasting blood glucose (mg/dl) | **-0.304** | **0.0005** |
| 2-hour postprandial glucose (mg/dl) | **-0.284** | **0.0012** |
| Total cholesterol (mg/dl) | 0.050 | 0.571 |
| Serum triglycerides (mg/dl) | -0.163 | 0.065 |
| HDL cholesterol (mg/dl) | **0.194** | **0.03** |
| LDL cholesterol (mg/dl) | 0.030 | 0.740 |
| MDA (nmol/ml) | 0.016 | 0.860 |
| GSH (mg/L) | 0.006 | 0.948 |

BMI, body mass index; GSH, glutathione; HbA1c, glycated hemoglobin; HDL, high-density lipoprotein; LDL, low-density lipoprotein; MDA, malondialdehyde; r, Spearman rank correlation coefficient

.**Supplementary Table S2. Correlation between plasma Vanin-1 and the studied parameters**.

|  | Correlation within the whole data | | Correlation within Q3 tertile | |
| --- | --- | --- | --- | --- |
|  | r | P | r | P |
| Age (year) | 0.078 | 0.379 | 0.155 | 0.398 |
| BMI (kg/m^2^) | -0.017 | 0.845 | 0.115 | 0.532 |
| HbA1c (%) | **0.2** | **0.023** | **0.451** | **0.01** |
| Fasting blood glucose (mg/dl) | 0.068 | 0.443 | 0.302 | 0.093 |
| 2-hour postprandial glucose (mg/dl) | 0.019 | 0.828 | 0.334 | 0.062 |
| Total cholesterol (mg/dl) | 0.089 | 0.319 | -0.346 | 0.247 |
| Serum triglycerides (mg/dl) | 0.025 | 0.728 | 0.263 | 0.146 |
| HDL cholesterol (mg/dl) | -0.072 | 0.418 | 0.217 | 0.234 |
| LDL cholesterol (mg/dl) | -0.029 | 0.747 | -0.314 | 0.080 |
| MDA (nmol/ml) | 0.078 | 0.379 | 0.024 | 0.897 |
| GSH (mg/L) | 0.016 | 0.859 | 0.162 | 0.348 |

BMI, body mass index; GSH, glutathione; HbA1c, glycated hemoglobin; HDL, high-density lipoprotein; LDL, low-density lipoprotein; MDA, malondialdehyde; r, Spearman rank correlation coefficient.

**Supplementary Table S3. Correlation between plasma reduced glutathione and the studied parameters.**

|  | r | P |
| --- | --- | --- |
| Age (year) | .062 | 0.486 |
| BMI (kg/m^2^) | 0.138 | 0.121 |
| HbA1c (%) | -0.010 | 0.910 |
| Fasting blood glucose (mg/dl) | -0.033 | 0.713 |
| 2-hour postprandial glucose (mg/dl) | -0.061 | 0.495 |
| Total cholesterol (mg/dl) | -0.027 | 0.766 |
| Serum triglycerides (mg/dl) | -0.017 | 0.847 |
| HDL cholesterol (mg/dl) | 0.076 | 0.223 |
| LDL cholesterol (mg/dl) | -0.016 | 0.858 |
| MDA (nmol/ml) | **0.223** | **0.011** |

BMI, body mass index; GSH, glutathione; HbA1c, glycated hemoglobin; HDL, high-density lipoprotein; LDL, low-density lipoprotein; MDA, malondialdehyde; r, Spearman rank correlation coefficient.

**Supplementary Table S4. Associations between antidiabetic medications and biomarkers levels**

| Biomarkers levels | Type of Therapy | | OR (95% confidence interval) | P-value |
| --- | --- | --- | --- | --- |
| Vanin-1 < 0.943ng/ml | **Insulin therapy** | 1.677 (0.33-4.63) | | 0.378 |
|  | **Metformin** | 0.660 (0.12-3.90) | | 0.660 |
|  | **Sulfonylureas** | 0.756 (0.184-3.12) | | 0.464 |
|  | **DPP-4 inhibitors** | 1.868 (0.35-9.96) | | 0.742 |
|  | **SGLT2 inhibitors** | 1.080(0.184-6.33) | | 0.932 |
| Pantothenic acid >1541.26 ng/ml | **Insulin therapy** | 1.022 (0.85-1.34) | | 0.447 |
|  | **Metformin** | 3.224 (0.37-28.19) | | 0.290 |
|  | **Sulfonylureas** | 0.392 (0.058-2.67) | | 0.340 |
|  | **DPP-4 inhibitors** | 0.233 (0.026-2.07) | | 0.191 |
|  | **SGLT2 inhibitors** | 0.508 (0.049-5.31) | | 0.572 |

DPP-4, dipeptidyl peptidase; OR, odds ratio; SGLT2, sodium glucose cotransporter
